# Working from home, work-time control and mental health: Results from the Brazilian longitudinal study of adult health (ELSA-Brasil)

**DOI:** 10.3389/fpsyg.2022.993317

**Published:** 2022-10-03

**Authors:** Rosane Harter Griep, Maria da Conceição C. Almeida, Sandhi Maria Barreto, André R. Brunoni, Bruce B. Duncan, Luana Giatti, José Geraldo Mill, Maria del Carmen B. Molina, Arlinda B. Moreno, Ana Luisa Patrão, Maria Inês Schmidt, Maria de Jesus Mendes da Fonseca

**Affiliations:** ^1^Laboratory of Health and Environmental Education, Instituto Oswaldo Cruz, Rio de Janeiro, Brazil; ^2^Instituto Gonçalo Moniz, Fundação Oswaldo Cruz, Salvador, Brazil; ^3^School of Medicine and Hospital das Clínicas/EBSERH, Universidade Federal de Minas Gerais, Belo Horizonte, Brazil; ^4^School of Medicine, Universidade de São Paulo, São Paulo, Brazil; ^5^Postgraduate Programme in Epidemiology and Hospital de Clínicas de Porto Alegre, Universidade Federal do Rio Grande do Sul, Porto Alegre, Brazil; ^6^Department of Physiological Sciences, Universidade Federal do Espírito Santo, Vitória, Brazil; ^7^Postgraduate Programme in Health and Nutrition, Universidade Federal do Ouro Preto, Ouro Preto, Brazil; ^8^Postgraduate Programme in Collective Health, Universidade Federal do Espírito Santo, Vitória, Brazil; ^9^Department of Epidemiology and Quantitative Methods in Health, Escola Nacional de Saúde Pública Sérgio Arouca, Fundação Oswaldo Cruz, Rio de Janeiro, Brazil; ^10^Center for Psychology, Faculty of Psychology and Education Science of the University of Porto, Porto, Portugal; ^11^Institute of Collective Health, Federal University of Bahia, Salvador, Brazil

**Keywords:** COVID-19, work from home, work-time control, mental health—related quality of life, stress

## Abstract

This cross-sectional study investigated the association between work-time control (WTC), independently and in combination with hours worked (HW), and four mental health outcomes among 2,318 participants of the Longitudinal Study of Adult Health (ELSA-Brasil) who worked from home during the COVID-19 pandemic. WTC was assessed by the WTC Scale, and mental health outcomes included depression, anxiety, stress (measured by the Depression, Anxiety and Stress Scale, DASS-21), and self-rated mental health. Logistic regression models were used to determine odds ratios (ORs) and 95% confidence intervals (CIs). Among women, long HW were associated with stress (OR = 1.56; 95% CI = 1.11–2.20) and poor self-rated mental health (OR = 1.64; 95% CI = 1.13–2.38), whereas they were protective against anxiety among men (OR = 0.59; 95% CI = 0.37–0.93). In both sexes, weak WTC was associated with all mental health outcomes. Among women, the long HW/weak WTC combination was associated with all mental health outcomes, and short HW/weak WTC was associated with anxiety and stress. Among men, long HW/strong WTC was protective against depression and stress, while short HW/strong WTC and short HW/weak WTC was associated with all mental health outcomes. In both sexes, weak WTC, independently and in combination with HW, was associated with all mental health outcomes. WTC can improve working conditions, protect against mental distress, and fosterwork-life balance for those who work from home.

## Introduction

In order to curb the spread of the disease caused by the 2019 coronavirus (COVID-19), prevent the collapse of health services, and reduce COVID-19 lethality, many countries introduced measures to restrict inter-person contact during the pandemic. These included guidance to stay at home, travel restrictions and closure of schools and non-essential services (Gostin and Wiley, 2020; Wenham et al., 2020; De Sio et al., 2021).

These measures led to unprecedented social distancing, with impacts on work organization and workers’ lives (Biroli et al., 2021). In several occupations, working from home became more frequent, necessary, and even mandatory. Workers had to try to perform their duties while also dealing with home-making, child-rearing, and various sources of distraction; often in very adverse ergonomic conditions and without any in-person interaction with co-workers (Arntz et al., 2020; Majumdar et al., 2020; Amano et al., 2021; Kniffin et al., 2021; Şentürk et al., 2021; Xiao et al., 2021).

Several studies have shown the adverse effects of these working arrangements on workers’ mental health during the COVID pandemic (Majumdar et al., 2020; Oakman et al., 2020; Biroli et al., 2021; Toniolo-Barrios and Pitt, 2021; Şentürk et al., 2021; Xiao et al., 2021). Working from home has been associated with psychosocial stress, social isolation, sleep disorders, concentration deficit, and screen fatigue from long working hours (Tavares, 2017; Majumdar et al., 2020; Buomprisco et al., 2021; Xiao et al., 2021). Long working hours have also been shown to have a significantly negative impact on worker’s psychological health (Virtanen et al., 2012; Watanabe et al., 2016; Li et al., 2019; Park et al., 2020), and have been identified as one of the pathways linking working from home and mental health (Choi et al., 2021; Rugulies et al., 2021; Toniolo-Barrios and Pitt, 2021; Şentürk et al., 2021). Indeed, those who work from home tend to work longer hours and spend more time on their cell phone and desktop/laptop than office workers (Nijp et al., 2016; Tavares, 2017; Majumdar et al., 2020). Working long hours can impact family activities and personal goals, foster imbalance between personal life and work (Żołnierczyk-Zreda et al., 2012), interfere with health-related behavior (Bannai and Tamakoshi, 2014; Virtanen et al., 2015) and reduce the time one has available for self-care (Soek et al., 2016). It appears that the challenge of transitioning to working from home is not the same for men and women. According to several studies (Arntz et al., 2020; Barbieri et al., 2021; Biroli et al., 2021; Sato et al., 2021; Şentürk et al., 2021; Xiao et al., 2021; Matthews et al., 2022) women’s mental health may be more severely impacted by this transition than men because of their greater involvement in household and caregiving tasks, which can cause work interruptions and concentration difficulties.

Studies prior to the pandemic indicated that giving workers control over their work schedules could attenuate the adverse mental health effects of working long hours (Ala-Mursula, 2002; Nijp et al., 2012; Żołnierczyk-Zreda et al., 2012). This kind of control allows workers to determine the length of their work day, when to start and finish their work, to take breaks and deal with private matters during work time, and to have the autonomy to schedule holidays and other kinds of leave (Nijp et al., 2012, 2015). It is based on workers’ needs rather than employers’ needs, and its positive effects stem from workers’ ability to balance their time and resources in order to better deal with the demands of work and home simultaneously (Żołnierczyk-Zreda et al., 2012; Leineweber et al., 2016; Virtanen et al., 2021).

As far as we were able to assess, no previous studies have explored the influence of control over working hours on mental health among individuals who worked from home during the COVID-19 pandemic. Furthermore, the literature has explored the effect of work control (Ala-Mursula, 2002; Albrecht et al., 2017; Li et al., 2019; Şentürk et al., 2021) and of long working hours from home (Park et al., 2020; Choi et al., 2021; Rugulies et al., 2021) on mental health separately. Besides, few articles have studied gender differences in vulnerability to the potential mental health effects of working from home (Matthews et al., 2022). In this article, we investigated gender differences in a comprehensive range of mental health outcomes, including depression (a mood disorder that involves a low mood and a loss of interest in activities), anxiety (a reaction to stress, with feelings of worry, nervousness, irritability or unease) and stress (a feeling of emotional or physical tension, caused by any event or thought that makes people feel worried, angry or nervous) (Sinclair et al., 2012; Vignola and Tucci, 2014; Bottesi et al., 2015; Camacho et al., 2016; Yıldırım et al., 2018; Martins et al., 2019). Beyond the experience of symptoms of depression, anxiety, and stress, overall self-rated measures can capture how people perceive their own mental health, rather than focusing on mental illness (Levinson and Kaplan, 2014), and can help improve screening and treatment interventions (McAlpine et al., 2018). Therefore, this study investigated the association between work-time control (WTC), independently and in combination with hours worked (HW), and depression, anxiety, stress and self-rated mental health among men and women who worked from home during the COVID-19 pandemic, highlighting sex differences.

## Materials and methods

### Sample and procedure

This cross-sectional study was carried out between July 2020 and February 2021, and used data from a supplementary study of the Longitudinal Study of Adult Health (*Estudo Longitudinal de Saúde do Adulto*, ELSA-Brasil) to assess the short- and long-term impacts of COVID-19 and of COVID-19 mitigation strategies. Within the framework of this supplementary study, 5,639 civil servants who were active in or retired from teaching positions and research institutions in five of Brazil’s state capitals (Belo Horizonte, Porto Alegre, Rio de Janeiro, Salvador, and Vitória) were invited to respond to questionnaires by mobile phone or computer. Invitees completed the questionnaires using an application that was produced especially for the study with the assistance of a trained, certified team. Only those who were active civil servants and responded to questions on working from home were eligible for inclusion (*n* = 3,043, 54%). We then excluded 725 individuals who did not engage in telework; thus the final analytical sample comprised 2,318 participants (1,155 men and 1,163 women).

### Measures

#### Hours worked from home

Hours worked (HW) was determined based on the question: “On average, how many hours do you spend on work at home, not counting housework?” Responses were categorized as “short HW” (< 12 h/week) and “long HW” (≥ 12 h/week), based on the median cut-off point for men and women.

#### Work-time control

Access to WTC was measured on the WTC access scale proposed by Nijp et al. (2015, 2016), who defined access to WTC as the possibility of deciding when to work. Three bilingual, epidemiological researchers with long experience in the psychometric adaptation of scales translated the WTC access scale from English to Portuguese. The instrument comprises six items, with responses given on a 5-point Likert scale (1 = never to 5 = always).

The dimensional validity of the WTC scale was assessed by exploratory and confirmatory factor analyses (EFA and CFA). In the EFA, the criteria used for the number of factors to be extracted were an eigenvalue greater than one and the factor structure fit, considering item loading and number of items per factor. Items with loading > 0.40 and no cross-loading (> 0.40 loading on more than one factor and < 0.20 difference between loadings), using the geomin oblique rotation, were considered appropriate. The CFA, based on the model originally proposed by the authors of the WTC scale (Nijp et al., 2015, 2016) and the results of the EFA, was then performed using the robust weighted least squares mean and variance adjusted (WLSMV) estimator. That analysis applied WLSMV, which uses polychoric correlation matrices as appropriate for categorical or ordinal variables. Model fit was assessed using the following criteria of proper fit: two incremental fit indices—the Comparative Fit Index (CFI) and Tuckey-Lewis Index (TLI) > 0.90—and one parsimonious fit index, RMSEA (< 0.06 preferable, but up to 0.08 acceptable) (Hu and Bentler, 1999; Hair et al., 2009). Convergent validity was assessed by average variance extracted (AVE) and internal consistency by composite reliability (CR), with AVE ≥ 0.50 and CR ≥ 0.60 considered acceptable (Hair et al., 2009). Cronbach’s alpha was also calculated in order to permit international comparisons. All analyses in this stage were performed using the Mplus statistical package, version 7.1 (Muthén and Muthén, 1998–2012). After assessing the WTC scale’s suitability by factor analysis, the sums of the scale items were calculated and median scores (women = 20; men = 21) were used to classify WTC as strong or weak.

Table 1 shows the items in English and in their Brazilian Portuguese translations, as well as the WTC scale’s final psychometric performance. The EFA revealed no cross-loading, and all items returned loadings of ≥ 40 (data not shown). The CFA showed satisfactory performance for all study indicators (CFI = 0.997, TLI = 0.998, RMSEA = 0.084), after inclusion of three residual correlations between pairs of items (1 and 2, 3 and 2, and 3 and 4). AVE and CR were 0.687 and 0.928, respectively (Table 1). This performance warranted proceeding with the analyses using the WTC scale and its association with the investigated mental health outcomes, both independently and in combination with HW.

**TABLE 1:** Items in the work time control scale, original version in English, version in Brazilian Portuguese, and results of the confirmatory factor analysis.

Items of the original scale^a^ worktime control access	Items of the scale translated into Brazilian Portuguese, as *acesso ao controle das horas de trabalho*	Confirmatory factor analysis	
		
		Factor loading	Residual variance
1. I control daily starting and ending times	1. Eu controlo diariamente a hora de começar e terminar meu trabalho	0.734	0.460
2. I control when to take a break	2. Eu controlo quando fazer um intervalo	0.729	0.469
3. I control when to take leave (day off or holiday)	3. Eu controlo quando tirar um dia de folga ou respeitar um feriado ou finais de semana	0.760	0.431
4. I control on which days to work	4. Eu controlo quais os dias em que trabalho	0.806	0.350
5. I control the distribution of work hours over the week	5. Eu controlo como distribuir minhas horas de trabalho durante a semana	0.956	0.090
6. I control my own working hours	6. Eu controlo minhas horas de trabalho	0.953	0.088
**Response categories—five-point Likert scale:** 1—(Almost) not at all; 2—to a limited extent; 3—to a reasonable extent; 4—to a high extent; and 5—to a very high extent	**Categorias de Respostas: escala Likert em cinco categorias:** 1-nunca, 2-raramente, 3-às vezes, 4-quase sempre, 5-sempre	**Indicators of fit** **Residual correlation:** **items 1 & 2; items 3 & 4;items 3 & 2**	
		CFI = 0.997; TLI = 0.998; RMSEA = 0.084; CR = 0.928; AVE = 0.687	

Supplementary study on COVID-19, ELSA-Brasil (2020–2021). ^a^Nijp et al., 2015, 2016.

CFI, Comparative Fit Index; TLI, Tucker Lewis Index; RMSEA, root mean square error of approximation; CR, composite reliability; AVE, average variance extracted.

### Combined variable hours worked/work time control

The combined variable HW/WTC was categorized at into four groups: short HW/strong WTC (reference category), long HW/strong WTC, short HW/weak WTC, and long HW/weak WTC.

### Depression, anxiety, and stress

Depression, anxiety and stress were evaluated by applying the Depression, Anxiety and Stress Scale—Short Form (DASS-21), translated into Brazilian Portuguese and validated (Vignola and Tucci, 2014). The DASS-21 comprises three subscales with seven items each, designed to evaluate depression (items: 3, 5, 10, 13, 16, 17, 21), anxiety (2, 4, 7, 9, 15, 19, 20) and stress (1, 6, 8, 11, 12, 14, 18) in the prior 7 days (Vignola and Tucci, 2014). Item responses are given on a 4-point Likert-type scale, ranging from 0 (“does not apply to me”) to 3 (“applies very much to me or most of the time”). Subscale scores were calculated by summing the item scores and multiplying by 2, so as to match the scoring on the original scale (DASS-42). Subscale scores were then categorized into five groups. For depression, group cut-offs were defined as normal (0–9 points), mild (10–13 points), moderate (14–20 points), severe (21–27 points) and extremely severe (≥ 28 points). For anxiety, they were normal (0–7 points), mild (8–9 points), moderate (10–14 points), severe (15–19 points) and extremely severe (≥ 20 points). Finally, for stress, the cut-offs were normal (0–14 points), mild (15–18 points), moderate (19–25 points), severe (26–33 points), and extremely severe (≥ 34 points) (Lovibond and Lovibond, 1995). For all these mental health outcomes, the categories mild and moderate, and severe and extremely severe, were collapsed into absent and present, respectively. The DASS-21 has been widely used and presented adequate, validated and reliable results in different samples in both international (Sinclair et al., 2012; Bottesi et al., 2015; Camacho et al., 2016; Yıldırım et al., 2018) and Brazilian (Vignola and Tucci, 2014; Martins et al., 2019) contexts. Cronbach’s alpha values were 0.88 for the depression subscale, 0.79 for the anxiety subscale and 0.88 for the stress subscale. In men, these values were 0.87, 0.74, and 0.87, respectively, and in women 0.89, 0.80, and 0.88, respectively.

#### Self-rated mental health

Self-rated mental health was assessed by the question: “Generally speaking, in comparison with people of your age, how do you regard your mental state of health?” Responses were grouped into “good” (very good/good) and “poor” (regular/poor/very poor). Self-rated mental health has been previously validated (Mawani and Gilmour, 2010) and is an important predictor of health outcomes and wellbeing (Ahmad et al., 2014; Levinson and Kaplan, 2014; McAlpine et al., 2018).

#### Covariables

Age (continuous in the multiple analysis and categorized as < 54 years, 55–64 years, ≥ 65 years in the bivariate analysis), self-reported race/color (black, white, brown, other), marital status (with partner, without partner), per capita income (continuous), schooling (masters/doctorate, undergraduate/higher diploma, up to complete upper secondary), caregiver for children, sick and/or elderly people (no, yes) and time spent on housework in hours/week (continuous) were included in the analyses as covariables.

#### Statistical analysis

In describing the sample, categorical variables were expressed as frequencies, and continuous variables as means and standard deviations (SDs). Associations were tested using the Pearson chi-square test for categorical variables and the *t*-test for continuous variables. Odds ratios (ORs) and their respective 95% confidence intervals (CIs) were estimated using logistic regression models, adjusted for covariables that showed associations in the bivariate analyses. Crude models included only the exposure and outcomes. Adjusted models included age, self-reported race/color, marital status, per capita income, and time spent on housework in hours/week. Interactions between sex and HW, WTC and HW/WTC were considered statistically significant at a *p*-value < 0.05. The descriptive analyses and analyses of association were stratified by sex and performed using the program R, version 3.6.1 (R Core Team, 2017).

## Results

Mean age in the study sample was 55 ± 7.4 years, and about half were women. The women were younger than the men, had less schooling (a smaller percentage held masters/doctoral degrees), reported lower income and more frequently reported caregiving responsibilities for children and the sick and/or elderly. However, men more frequently reported having a partner. Mean HW (20.5 ± 17.7 for women and 20.6 ± 17.9 for men), as well as the frequencies of different HW/WTC groups, were similar in both sexes. On the other hand, women reported spending more time on housework than men and displayed higher prevalences of all mental health outcomes (Table 2).

**TABLE 2:** Description of study variables, by sex.

Sample descriptive variables	Women (*N* = 1,163)		Men (*N* = 1,155)	
Age (years) mean ± SD*	54.9	7.3	55.5	7.5
**Schooling** *n* (%)***				
Masters/Doctorate	588	51.0	657	57.3
Undergraduate/Higher diploma	443	38.5	342	29.8
Secondary schooling or less	121	10.5	147	12.8
Per capita income mean ± SD**	5020.7	3533.7	4652.1	2943.7
**Self-reported race/color** *n* (%)				
White	699	61.9	691	61.2
Brown	291	25.8	331	29.3
Black	123	10.9	90	8.0
Other (indigenous and yellow)	17	1.5	17	1.5
Marital status—with partner *n* (%)***	522	51.4	800	80.8
Cares for children, sick and/or elderly persons *n* (%)*	397	39.1	345	34.8
Hours housework per week mean ± SD**	15.0	16.3	11.1	14.5
Hours working from home per week mean ± SD	20.6	17.9	20.5	17.7
Long hours working from home per week (HW) *n* (%)	587	50.9	577	50.2
Strong work time control (WTC) *n* (%)	547	47.0	527	45.6
**Combination of HW and WTC** *n* (%)				
Short HW/Strong WTC	324	28.1	330	28.1
Long HW/Strong WTC	287	24.9	297	25.8
Short HW/Weak WTC	242	21.0	242	21.1
Long HW/Weak WTC	300	26.0	280	24.4
Poor self-rated mental health *n* (%)*	185	16.0	148	13.0
Depression *n* (%)***	286	24.7	212	18.6
Anxiety *n* (%)***	237	20.5	121	10.6
Stress *n* (%)***	233	20.1	134	11.7

Supplementary study on COVID-19, ELSA-Brasil (2020–2021). *p < 0.05; **p < 0.01; ***p < 0.001.

Among women, long HW (≥ 12 h/week) was associated with stress (OR = 1.56, 95% CI = 1.11–2.20) and poor self-rated mental health (OR = 1.64, 95% CI = 1.13–2.38). However, among men, long HW proved protective for anxiety (OR = 0.59, 95% CI = 0.38–0.91). In both sexes, strong WTC was negatively associated with all mental health outcomes (Table 3).

**TABLE 3:** Crude and adjusted associations (OR 95%CI) between hours worked from home (HW), work time control (WTC), and mental health outcomes.

		Depression	Anxiety	Stress	Poor self-rated mental health

Hours working from home (HW)					
women					
Crude model	Short HW	1	1	1	1
	Long HW	**1.39 (1.06;1.82)**	1.31 (0.98; 1.75)	**1.64 (1.22;2.20)**	**1.70 (1.23;2.35)**
	AIC	1285.0	1167.1	1148.4	1004.0
Adjusted model^a^	Short HW	1	1	1	1
	Long HW	1.30 (0.96;1.78)	1.28 (0.92;1.79)	**1.56 (1.11;2.20)**	**1.64 (1.13;2.38)**
	AIC	1080.1	965.4	944.5	836.8

			**Men**		

Crude model	Short HW	1	1	1	1
	Long HW	0.97 (0.72;1.30)	0.79 (0.54;1.14)	0.86 (0.60;1.23)	0.91 (0.65;1.29)
	AIC	1094.5	773.02	823.64	882.81
Adjusted model^a^	Short HW	1	1	1	1
	Long HW	0.89 (0.63;1.26)	**0.59 (0.37;0.93)**	0.75 (0.49;1.14)	0.98 (0.67;1.45)
	AIC	890.8	608.2	663.3	748.2

**Work time control (WTC)**					
**women**					

Crude model	Alto WTC	1	1	1	1
	Weak WTC	**1.43 (1.09;1.87)**	**1.87 (1.40; 2.50)**	**2.06 (1.53;2.76)**	**1.54 (1.12;2.12)**
	AIC	1291.8	1159.5	1143.1	1014.1
Adjusted model^a^	Strong WTC	1	1	1	1
	Weak WTC	**1.40 (1.04;1.88)**	**2.14 (1.55;2.97)**	**2.28 (1.63;3.17)**	**1.68 (1.18;2.38)**
	AIC	1085.0	952.6	932.6	841.6

			**Men**		

Crude model	Strong WTC	1	1	1	1
	Weak WTC	**2.02 (1.49;2.74)**	**1.87 (1.27;2.74)**	**3.31 (2.24;4.91)**	**2.09 (1.46–2.97)**
	AIC	1078.8	765.58	790.53	867.71
Adjusted model^a^	Strong WTC	1	1	1	1
	Weak WTC	**2.35 (1.66;3.33)**	**2.03 (1.30–3.15)**	**3.61 (2.31;5.65)**	**2.19 (1.48;3.24)**
	AIC	871.2	604.9	635.6	733.6

Supplementary study on COVID-19, ELSA-Brasil (2020–2021). *^a^*Adjusted for age, income, marital status, self-reported race/color, and hours of housework per week.

Statistically significant values in bold.

Results for HW/WTC groups differed for men and women. Among women, when compared with short HW/strong WTC, short HW/weak WTC was associated with anxiety (OR = 1.82, 95% CI = 1.12–2.97) and stress (OR = 1.67, 95% CI 1.01–2.78), while long HW/weak WTC was associated with all mental health outcomes: depression (OR = 1.76, 95% CI = 1.16–2.65); anxiety (OR = 2.51, 95% CI = 1.59–3.95); stress (OR = 3.16, 95% CI = 1.99–5.03) and poor self-reported mental health (OR = 2.48, 95% CI = 1.51–4.05). Among men, long HW/strong WTC was protective against depression (OR = 0.56, 95% CI = 0.33–0.97) and stress (OR = 0.37, 95%CI = 0.16–0.85), and short HW/weak WTC was associated with depression, anxiety and stress (respectively, OR = 1.67, 95% CI = 1.04–2.68; OR = 1.93, 95% CI = 1.1−03.36; and OR = 2.57, 95% CI = 1.46–4.54). Finally men in the long HW/weak WTC group had higher odds of depression, stress and poor self-rated mental health than those in the short HW/strong WTC group (respectively, OR = 2.10, 95% CI = 1.31–3.38; OR = 2.60, 95% CI = 1.44–4.68; and OR = 2.11, 95% CI = 1.24–3.58) (Table 4).

**TABLE 4:** Crude and adjusted associations (OR 95% CI) between hours worked from home (HW) and work time control (WTC) combined and mental health outcomes.

		Depression	Anxiety	Stress	Poor self-rated mental health
		**Women**			
	
Crude model	Short HW and strong WTC	1	1	1	1
	Long HW and strong WTC	1.14 (0.77;1.67)	1.09 (0.71;1.69)	1.12 (0.71;1.75)	1.21 (0.76; 1.94)
	Short HW and weak WTC	1.17 (0.78;1.75)	**1.60 (1.04;2.46)**	1.45 (0.92;2.27)	1.07 (0.65;1.77)
	Long HW and weak WTC	**1.87 (1.30;2.69)**	**2.23 (1.51;3.31)**	**2.93 (1.97;4.36)**	**2.33 (1.52;3.56)**
	AIC	1281.1	1153.8	1126.4	998.48
Adjusted model^a^	Short HW and strong WTC	1	1	1	1
	Long HW and strong WTC	1.09 (0.71;1.67)	1.07 (0.65;1.75)	1.11 (0.66;1.85)	1.19 (0.70;2.03)
	Short HW and weak WTC	1.17 (0.75;1.83)	**1.82 (1.12;2.97)**	**1.67 (1.01;2.78)**	1.20 (0.68;2.11)
	Long HW and weak WTC	**1.76 (1.16;2.65)**	**2.51 (1.59;3.95)**	**3.16 (1.99;5.03)**	**2.48 (1.51;4.05)**
	AIC	1078.1	948.7	922.4	830.6
	
		**Men**			
	
Crude model	Short HW and strong WTC	1	1	1	1
	Long HW and strong WTC	0.65 (0.40; 1.04)	0.75 (0.42;1.36)	**0.43 (0.21;0.89)**	0.57 (0.32;1.01)
	Short HW and weak WTC	**1.55 (1.02;2.36)**	**1.91 (1.14;3.19)**	**2.46 (1.48;4.11)**	1.55 (0.96;2.50)
	Long HW and weak WTC	**1.87 (1.25;2.79)**	1.45 (0.86;2.45)	**2.52 (1.53;4.14)**	**1.78 (1.13;2.81)**
	AIC	1071.9	765.81	781.25	865.4
Adjusted model^a^	Short HW and strong WTC	1	1	1	1
	Long HW and strong WTC	**0.56 (0.33;0.97)**	0.54 (0.27;1.08)	**0.37 (0.16;0.85)**	0.62 (0.33;1.14)
	Short HW and weak WTC	**1.67 (1.04;2.68)**	**1.93 (1.10;3.36)**	**2.57 (1.46;4.54)**	1.52 (0.89;2.59)
	Long HW and weak WTC	**2.10 (1.31;3.38)**	1.22 (0.65;2.30)	**2.60 (1.44;4.68)**	**2.11 (1.24;3.58)**
	AIC	864.4	601.8	626.1	731.9

Supplementary study on COVID-19, ELSA-Brasil (2020–2021). *^a^*Adjusted for age, income, marital status, self-reported race/color, and hours of housework per week.

Statistically significant values in bold.

Significant interactions were found between long HW and anxiety (*p* = 0.00187) and stress (*p* = 0.00871), weak WTC and depression (*p* = 0.01648), strong WTC/long HW and depression (*p* = 0.043563), weak WTC/long HW and anxiety (*p* = 0.030243) and between strong WTC/long HW and stress (*p* = 0.005665).

## Discussion

This study examined sex differences in the association between WTC, independently and in combination with HW, and depression, anxiety, stress, and self-rated mental health among individuals who worked from home during the COVID-19 pandemic. Moreover, it is one of the first studies to provide evidence of the modifying effect of the combination of WTC and HW on mental health outcomes in this group of workers. The study showed that, among people working from home, long HW (i.e., above median hours) increased the odds of stress and of poor self-rated mental health among women, while among men, the direction of the association indicated protection against anxiety. Weak WTC was associated with all mental health outcomes in both sexes, independently and in combination with short and long HW. The high prevalence of depression, anxiety and stress observed in the present study, especially among women, is in agreement with other studies carried out during the pandemic (Şentürk et al., 2021; Wang et al., 2021; Andersen et al., 2022).

Men’s and women’s HW were similar, but medians were lower than those reported in other studies, in which men were shown to have a longer work day (Li et al., 2019; Park et al., 2020; Choi et al., 2021; Rugulies et al., 2021); these include previous studies conducted as part of ELSA-Brasil. Also, the sex differences in the associations between HW and the mental health outcomes showed a pattern of interaction, with long HW being associated with higher odds of mental health outcomes among women, but conferring a protective effect among men. This pattern was also observed in a subgroup analysis (Choi et al., 2021) investigating the association between working 41–52 h/week and mild depression and moderate to severe depression, although the estimates for men were not statistically significant, and the same study showed an association between long work weeks and the risk of stress, depression and suicidal ideas, with more prominent associations observed among women and low-wage workers (Choi et al., 2021).

A recent review (Rugulies et al., 2021) concluded that there is still insufficient evidence on the association between HW and mental health outcomes, and underlined the need for further studies. However, most studies on this topic address the situation prior to the pandemic and do not take into account work-from-home factors in the context of the COVID-19 pandemic. It is possible that HW during the pandemic, even if they were shorter than what would have been routine before the pandemic, are still reflected in women’s mental health. Indeed, the pandemic had a strong impact on family life (Arntz et al., 2020; Biroli et al., 2021) and imposed new routines that placed different demands on women. For example, our findings showed that a higher proportion of women had no partner, had to care for children or a sick family member and had a higher average number of hours of housework. A study of families in the United States, Britain and Italy (Biroli et al., 2021) showed that women reported spending more time on household chores during than before the COVID-19 pandemic, and that men reported taking greater part in caring for children (especially in play time) and in grocery shopping. It is therefore possible that women find it more difficult to work from home, because they tend to spend more time on a variety of household and caregiving tasks, and thus may face more frequent interruptions at work, difficulty concentrating and lack of support in housework (Arntz et al., 2020; Biroli et al., 2021; Şentürk et al., 2021; Xiao et al., 2021). A qualitative study in Turkey even found that telework can detach women from professional work, expose them to more precarious labor conditions and consolidate their roles as traditional housewives (Çoban, 2021). Barbieri et al. (2021) found a slightly greater effect of workload on women than on the overall sample. The authors emphasized the view that women’s professional life is considered complementary, whereas the domestic avenue is given higher priority. In line with the present study, others (Sato et al., 2021; Şentürk et al., 2021; Xiao et al., 2021) have indicated that women working from home were more likely to suffer worse mental health outcomes than men. These findings suggest that working from home can heighten gender inequalities in various dimensions.

The importance of WTC and its association with mental health outcomes differed slightly between the sexes. In both men and women, weak WTC was associated with anxiety and stress, even when combined with short HW. However, stronger associations were observed when long HW and weak WTC were present simultaneously. WTC was found to be protective among men, even when combined with long HW, thus reinforcing the importance of WTC. Previous studies have also pointed to the importance of WTC, especially when working from home (Li et al., 2019, as cited in de Wind et al., 2021), saying that WTC fosters balance between family life and work, motivation to work, physical and mental health, and reduces fatigue (Nijp et al., 2012, 2015, 2016; Albrecht et al., 2017, 2020). Other studies (Ala-Mursula, 2002; Nijp et al., 2012; Żołnierczyk-Zreda et al., 2012; Leineweber et al., 2016) have found that, although WTC is universally beneficial, workers with more family responsibilities (especially women with small children) and those with more need to recover after working (older workers), are more likely to derive greater benefit from increased WTC, even when working longer hours.

Working from home, an arrangement that is increasingly common worldwide, can be beneficial to both workers and employers, especially workers who live in large cities, as they eliminate commuting time, and workers with motor deficiencies (Tavares, 2017; Majumdar et al., 2020; Buomprisco et al., 2021; Conroy et al., 2021; Xiao et al., 2021). However, it can increase the costs workers incur to create the proper infrastructure for work and deprive workers of interpersonal relations with fellow workers. In the context of the pandemic, the professional isolation produced by social distancing contributed to burnout and stress (Jamal et al., 2021). Some groups, especially women with small children, may find their workload increased by the combination of housework and job demands, which can produce stress and impair work performance (Ala-Mursula, 2002; Nijp et al., 2012; Żołnierczyk-Zreda et al., 2012; Leineweber et al., 2016). Also, it is important to emphasize that working from home during the pandemic differs from conventional telework in that, when it is mandatory to work from home full-time, some of the advantages of conventional telework, such as flexibility in working hours, in the workplace and in the respect for workers’ preferences in general, may not be maintained (Barbieri et al., 2021).

Moreover, that the extensive use of information and communication technologies (ICT) when working from home can result in “techno-overload” (Ragu-Nathan et al., 2008). This overload is considered a techno-stressor which, when combined with stressful situations, contributes to a longer and faster-paced working day, possibly involving handling large amounts of information, leading to fatigue, memory difficulties and loss of WTC. Also, some workers may have difficulty dealing with all the skills and know-how relating to new ICT updates, which in turn can cause greater pressure and tension (Ingusci et al., 2021).

The main strengths of the present study lie in the adaptation of the WTC scale to Brazilian Portuguese, with psychometric properties appropriate to a relatively large, wide-ranging sample, comprising personnel drawn from a variety of professions at different universities. Another strength is the use of the variables HW and WTC independently and in combination. Moreover, in addition to the use of three different mental health outcomes assessed by the DASS-21 (depression, anxiety, and stress), we used self-rated mental health as an outcome. As many mental health conditions remain undiagnosed, self-rated assessments provide a useful and perhaps more revealing indicator of mental wellbeing (Levinson and Kaplan, 2014; McAlpine et al., 2018), and may capture a more comprehensive understanding of mental health in the general population (Mawani and Gilmour, 2010; Nguyen et al., 2015; Romac et al., 2022). Self-rated mental health is also qualitatively different from mental illness, as it goes beyond the experience of symptoms (Levinson and Kaplan, 2014), and it is related to health care expenditure (Nguyen et al., 2015).

Limitations of the study include the lack of representativeness in the sample, which was obtained by voluntary completion of questionnaires *via* an application software. That strategy, which was quite common during the pandemic, may have led to self-selection bias, particularly among participants with more schooling, as observed in this and other studies (Amano et al., 2021; Barbieri et al., 2021). However, it is important to emphasize that this was expected, given that working from home has historically been the privilege of those in better socioeconomic positions (Wang et al., 2021). Another limitation is the cross-sectional nature of the analyses, in that exposure and outcome were obtained at the same time, thus the possibility of reverse causality cannot be ruled out, and participants in mental distress may have perceived weaker WTC and longer HW. However, some authors have already demonstrated that the direction of the effect is predominantly from WTC to the subsequent mental health outcomes (Albrecht et al., 2017, 2020). The occupations of the participants were not included in the study, and this variable may influence HW. Another limitation is that the long-term effects of social distancing measures on telework have not been evaluated, and the analyses presented here covered early stages of the pandemic.

One of the major challenges posed by the COVID-19 pandemic has been the sudden change in how people and their families live, work, study, and carry out their daily routines. Working from home has gone from being an occasional activity to a permanent, constant feature of the domestic environment. In addition, this work is being done full-time, rather than part-time or occasionally, and can have harmful effects on wellbeing and stress levels. Understanding how telework is organized and its impacts on mental health, especially in a new context such as that presented during the pandemic, will make it possible to develop strategies and public policies to protect workers’ health. The findings of this study point to the importance of strengthening WTC, a strategy that should be pursued widely among teleworkers. This study can inform recommendations to managers and workers in connection with for telework, an increasingly common arrangement in the service sector, particularly with regard to the introduction of rest periods, promoting leisure activities and respecting the working hours that are best suited to each individual’s demands and productivity.

Future studies should be carried out that include the role of housework and the constitution of families (number of members, type of household, etc.) among the examined associations. These variables can complement the study of the effects of working from home on workers’ mental health.

## Data availability statement

The datasets presented in this article are not readily available because the ELSA study has government funding and the database is available only to researchers and students of the research institutions linked to the study. Requests to access the datasets should be directed to elsa@fiocruz.br.

## Ethics statement

The studies involving human participants were reviewed and approved by the research ethics committees of all five researchers centers (Federal University of Minas Gerais, Federal University of Rio Grande do Sul, Federal University of Espírito Santo, Federal University of Bahia, and Oswaldo Cruz Foundation). The participants provided their written informed consent to participate in this study.

## Author contributions

RG coordinated the study design, wrote the manuscript, and had primary responsibility for the final content. MA, SB, BD, LG, JM, MM, MS, and MF coordinated the study design, participated in data interpretation, contributed intellectual content to the manuscript, and participated in final review of the manuscript. AB, AM, and AP participated in data interpretation, contributed intellectual content to the manuscript, and participated in final review of the manuscript. All authors have read and approved the final version of manuscript.
